# Supplementary figures and images for: Species-specific thermal classification schemes can improve climate related marine resource decisions
Source: PLoS One. 2021 Apr 28;16(4):e0250792. doi: 10.1371/journal.pone.0250792 (PMC8081253; doi:10.1371/journal.pone.0250792)

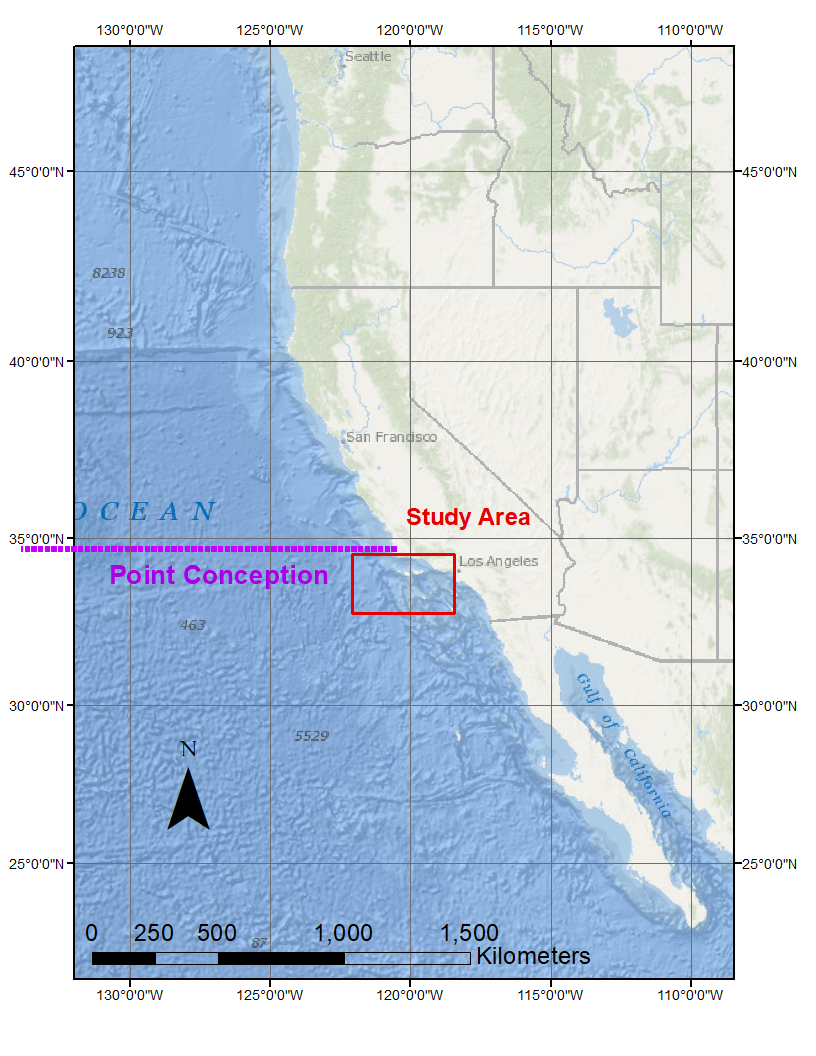

Supplement: S1 Fig — This map depicts the biogeographic break of Point Conception (shown in purple) in relation to the study area (bounded by the red box) where data was collected to assess ecosystem response to the marine heatwave. (TIF) [file pone.0250792.s001.tif]
