# Supplementary material for: Species-specific thermal classification schemes can improve climate related marine resource decisions
Source: PLoS One. 2021 Apr 28;16(4):e0250792. doi: 10.1371/journal.pone.0250792 (PMC8081253; doi:10.1371/journal.pone.0250792)
Supplement: S1 Table — The classifications of species (n = 152) are shown below across data sources and their resulting composite classification and agreement ranking. Species below the second black line are found in the recruitment dataset but not the diver survey data. Blank spaces denote that data was not available to create a classification and species are unclassified. (DOCX) [file pone.0250792.s002.docx]

**S1 Table. The species classification methods**. The classifications of species (n= 152) are shown below across data sources and their resulting composite classification and agreement ranking. Species below the second black line are found in the recruitment dataset but not the diver survey data. Blank spaces denote that data was not available to create a classification and species are unclassified.

|  |  |  | **Expert Opinions** | **Range Limits** | **Museum Collection** | **Diver Survey** | **Composite Classification** | **Agreement** |
| --- | --- | --- | --- | --- | --- | --- | --- | --- |
|  |  | **Warm Species** | 44 (28.9%) | 59 (38.8%) | 56 (36.8%) | 44 (28.9%) | 59 (38.8%) | High = 78 |
|  |  | **Cool Species** | 77 (50.6%) | 83 (54.6%) | 62 (40.7%) | 32 (21%) | 74 (48.7%) | Moderate = 27 |
|  |  | **Eurythermal Species** | 29 (19.0%) | 1 (0.6%) | 21 (13.8%) | 15 (9.9%) | 19 (12.5%) | Low = 47 |
|  |  | **Unclassified Species** | 2 (1.3%) | 9 (5.9%) | 13 (8.5%) | 61 (40.1%) |  |  |
| **Genus** | **Species** | **Common Name** | **Expert Opinions** | **Range Limits** | **Museum Collection** | **Diver Survey** | **Composite Classification** | **Agreement** |
| Zapteryx | exasperata | Banded Guitarfish | Warm | Warm | Warm |  | Warm | High |
| Paralabrax | nebulifer | Barred Sandbass | Warm | Warm | Warm | Warm | Warm | High |
| Amphistichus | argeneus | Barred Surfperch | Warm | Warm | Warm |  | Warm | High |
| Myliobatis | californica | Bat Ray | Warm | Warm | Eurythermal | Warm | Warm | Moderate |
| Lepidogobius | lepidus | Bay Goby | Cool | Cool | Cool |  | Cool | Low |
| Sebastes | chrysomelas | Black and Yellow Rockfish | Cool | Warm | Eurythermal | Cool | Cool | Low |
| Cheilotrema | saturnum | Black Croaker | Warm | Warm | Warm | Warm | Warm | High |
| Sebastes | melanops | Black Rockfish | Cool | Cool | Cool | Cool | Cool | High |
| Embiotoca | jacksoni | Black Surfperch | Cool | Warm | Eurythermal | Warm | Warm | Low |
| Rhinogobiops | nicholsii | Blackeye Goby | Warm | Cool | Warm | Cool | Eurythermal | Low |
| Chromis | punctipinnis | Blacksmith | Warm | Warm | Warm | Warm | Warm | High |
| Sebastes | mystinus | Blue Rockfish | Cool | Cool | Cool | Cool | Cool | High |
| Prionace | glauca | Blue Shark | Eurythermal | Warm | Warm | Eurythermal | Warm | Low |
| Lythrypnus | dalli | Bluebanded Goby | Warm | Warm | Warm |  | Warm | High |
| Sebastes | paucispinis | Bocaccio | Cool | Cool | Cool | Eurythermal | Cool | Moderate |
| Notorynchus | cepedianus | Broadnose Sevengill Shark | Eurythermal | Eurythermal | Cool |  | Eurythermal | Low |
| Sebastes | auriculatus | Brown Rockfish | Cool | Cool | Cool | Cool | Cool | High |
| Enophrys | bison | Buffalo Sculpin | Cool | Cool | Cool | Cool | Cool | High |
| Plueronichthys | coenosus | C-O Turbot | Cool | Cool |  |  | Cool | Moderate |
| Scorpaenichthys | marmoratus | Cabezon | Eurythermal | Cool | Cool | Cool | Cool | Moderate |
| Sebastes | dalli | Calico Rockfish | Warm | Warm | Warm | Warm | Warm | High |
| Sphyraena | argentea | California Barracuda | Warm | Cool | Warm | Warm | Warm | Moderate |
| Paralichthys | californicus | California Halibut | Warm | Cool | Warm | Warm | Warm | Moderate |
| Synodus | lucioceps | California Lizardfish | Warm | Warm | Warm | Warm | Warm | High |
| Gymnothorax | mordax | California Moray | Warm | Warm | Warm | Warm | Warm | High |
| Xenistius | californiensis | California Salema | Warm | Warm | Warm |  | Warm | High |
| Scorpaena | guttata | California Scorpionfish | Warm | Warm | Warm | Warm | Warm | High |
| Semicossyphus | pulcher | California Sheephead | Warm | Warm | Warm | Warm | Warm | High |
| Sebastes | pinniger | Canary Rockfish | Cool | Cool | Cool | Cool | Cool | High |
| Sebastes | nebulosus | China Rockfish | Cool | Cool | Cool | Cool | Cool | High |
| Sebastes | caurinus | Copper Rockfish | Cool | Cool | Cool | Cool | Cool | High |
| Artedius | corallinus | Coralline Sculpin | Cool | Cool | Eurythermal |  | Cool | Moderate |
| Mircometrus | minimus | Dwarf Surfperch | Warm | Warm | Warm | Warm | Warm | High |
| Sarda | chiliensis | Eastern Pacific Bonito | Warm | Warm | Warm | Warm | Warm | High |
| Balistes | polylepis | Finescale Triggerfish | Warm | Warm | Warm | Warm | Warm | High |
| Sebastes | rubrivinctus | Flag Rockfish | Cool | Cool | Cool |  | Cool | High |
| Hypsypops | rubicundus | Garibaldi | Warm | Warm | Warm | Warm | Warm | High |
| Heterostichus | rostratus | Giant Kelpfish | Cool | Cool | Warm | Warm | Eurythermal | Low |
| Stereolepis | gigas | Giant Sea Bass | Warm | Cool | Warm | Warm | Warm | Moderate |
| Sebastes | carnatus | Gopher Rockfish | Cool | Warm | Warm | Cool | Eurythermal | Low |
| Sebastes | rastrelliger | Grass Rockfish | Cool | Cool | Eurythermal | Eurythermal | Cool | Low |
| Atherinopsidae | spp. | Grunion, Topsmelt or Jacksmelt | Warm |  |  | Warm | Warm | High |
| Rhamphocottus | richardsoni | Grunt sculpin | Cool | Cool | Cool |  | Cool | High |
| Apogon | guadalupensis | Guadalupe Cardinalfish | Warm | Warm | Warm |  | Warm | High |
| Sebastes | semicinctus | Halfbanded Rockfish | Warm | Warm | Warm | Warm | Warm | High |
| Medialuna | californiensis | Halfmoon | Warm | Warm | Warm | Warm | Warm | High |
| Sebastes | umbrosus | Honeycomb Rockfish | Warm | Warm | Warm |  | Warm | High |
| Heterodontus | francisci | Horn Shark | Eurythermal | Warm | Warm | Warm | Warm | Moderate |
| Alloclinus | holderi | Island Kelpfish | Warm | Warm | Warm | Warm | Warm | High |
| Trachurus | symmetricus | jack Mackerel | Eurythermal | Cool | Warm | Warm | Warm | Low |
| Atherinopsis | californiensis | Jacksmelt | Eurythermal | Cool | Eurythermal |  | Eurythermal | Low |
| Paralabrax | clathratus | Kelp Bass | Eurythermal | Cool | Warm | Warm | Warm | Low |
| Lethops | connectens | Kelp Goby | Cool | Warm | Warm |  | Warm | Moderate |
| Hexagrammos | decagrammus | Kelp Greenling | Cool | Cool | Cool | Cool | Cool | High |
| Apodichthys | sanctaerosae | Kelp Gunnel | Cool | N/A |  |  | Cool | Low |
| Sebastes | atrovirens | Kelp Rockfish | Cool | Warm | Warm | Eurythermal | Warm | Low |
| Brachyistius | frenatus | Kelp Surfperch | Cool | Cool | Cool | Eurythermal | Cool | Moderate |
| Gibbonsia | spp. | Kelpfish |  |  |  | Cool | Cool | Low |
| Clinidae | spp. | Kelpfishes and Fringeheads |  |  |  | Warm | Warm | Low |
| Leiocottus | hirundo | Lavender Sculpin | Cool | Warm | Warm |  | Warm | Low |
| Triakis | semifasciata | Leopard Shark | Eurythermal | Warm | Eurythermal |  | Eurythermal | Low |
| Mircometrus | elongatus | Lingcod | Cool | Cool | Cool | Cool | Cool | High |
| Jordania | zonope | Longfin Sculpin | Cool | Cool | Cool | Cool | Cool | High |
| Ernogrammus | walkeri | Masked Prickleback | Cool | Cool | Cool |  | Cool | High |
| Cebidichthys | violaceus | Monkeyface Eel | Cool | Cool | Cool | Cool | Cool | High |
| Chirolophis | nugator | Mosshead Warbonnet | Cool | Cool | Cool |  | Cool | High |
| Engraulis | mordax | Northern Anchovy | Eurythermal | Cool | Cool | Eurythermal | Cool | Low |
| Gobiesox | maeandricus | Northern Clingfish | Cool | Cool | Cool |  | Cool | High |
| Ronquilus | jordani | Northern Ronquil | Cool | Cool | Cool |  | Cool | High |
| Mola | Mola | Ocean Sunfish | Eurythermal | Warm | Eurythermal |  | Eurythermal | Low |
| Caulolatilus | princeps | Ocean Whitefish | Warm | Warm | Warm |  | Warm | High |
| Sebastes | serranoides,flavidus | Olive or Yellowtail Rockfish | Cool | N/A |  | Eurythermal | Eurythermal | Low |
| Neoclinus | uninotatus | Onespot Fringehead | Cool | Cool | Eurythermal |  | Cool | Low |
| Girella | nigricans | Opaleye | Warm | Warm | Warm | Warm | Warm | High |
| Squatina | californica | Pacific Angel Shark | Eurythermal | Cool | Warm | Eurythermal | Eurythermal | Low |
| Torpedo | californica | Pacific Electric Ray | Warm | Cool | Cool | Cool | Cool | Moderate |
| Scomber | japonicus | Pacific Mackerel | Cool | Warm | Eurythermal | Warm | Warm | Low |
| Citharichthys | sordidus | Pacific Sanddab | Cool | Cool | Cool |  | Cool | High |
| Sardinops | sagax | Pacific Sardine | Eurythermal | Cool | Eurythermal | Warm | Eurythermal | Low |
| Oxylebius | pictus | Painted Greenling | Cool | Cool | Cool | Eurythermal | Cool | Moderate |
| Rhacochilus | vacca | Pile Surfperch | Cool | Cool | Cool | Warm | Cool | Moderate |
| Zalembius | rosaceus | Pink Surfperch | Eurythermal | Warm | Eurythermal |  | Eurythermal | Low |
| Porichthys | notatus | Plainfin Midshipman | Cool | Cool | Cool |  | Cool | High |
| Sebastes | maliger | Quillback rockfish | Cool | Cool | Cool | Cool | Cool | High |
| Hypsurus | caryi | Rainbow Surfperch | Cool | Cool | Eurythermal | Eurythermal | Cool | Low |
| Hemilepidotus | hemilepidotus | Red Irish Lord | Cool | Cool | Cool | Cool | Cool | High |
| Hexagrammos | lagocephalus | Rock Greenling | Cool | Cool | Cool | Cool | Cool | High |
| Halicoeres | semicinctus | Rock Wrasse | Warm | Warm |  | Warm | Warm | High |
| Sebastes | rosaceus | Rosy Rockfish | Cool | Cool | Cool | Cool | Cool | High |
| Chitonotus | pugetensis | Roughback Sculpin | Eurythermal | Cool | Cool |  | Cool | Moderate |
| Urobatis | halleri | Round Ray | Warm | Warm | Warm |  | Warm | High |
| Rhacochilus | toxotes | Rubberlip Surfperch | Warm | Warm | Eurythermal | Warm | Warm | Moderate |
| Neoclinus | blanchardi | Sarcastic Fringehead | Eurythermal | Warm | Warm |  | Warm | Low |
| Clupeidae | spp. | Sardines and Anchovies | Warm | N/A |  |  | Warm | Low |
| Anisotremus | davidsonii | Sargo | Warm | Warm | Warm | Warm | Warm | High |
| Chaetodon | falcifer | Scythemarked Butterflyfish | Warm | Warm | Warm |  | Warm | High |
| Oxyjulis | californica | Senorita | Warm | Warm | Warm | Warm | Warm | High |
| Phanerodon | atripes | Sharpnose Surfperch | Cool | Warm | Eurythermal |  | Eurythermal | Low |
| Cymatogaster | aggregata | Shiner Surfperch | Cool | Cool | Cool | Warm | Cool | Moderate |
| Rhinobatos | productus | Shovelnose Guitarfish | Warm | Warm | Warm |  | Warm | High |
| Hyperprosopon | ellipticum | Silver Surfperch | Cool | Cool | Cool | Cool | Cool | High |
| Kasatkia | seigeli | Six-spot Prickleback | Cool | N/A |  |  | Cool | Low |
| Rathbunella | hypoplecta | Smooth Ronquil | Eurythermal | Cool | Warm | Eurythermal | Eurythermal | Low |
| Orthonopias | triacis | Snubnose Sculpin | Cool | Warm | Eurythermal |  | Eurythermal | Low |
| Citharichthys | stigmaeus | Speckled Sanddab | Cool | Cool | Cool |  | Cool | High |
| Squalus | acanthias | Spiny Dogfish | Cool | Cool | Cool | Eurythermal | Cool | Moderate |
| Sebastes | diploproa | Spitnose Rockfish | Cool | Cool | Cool |  | Cool | High |
| Hyperprosopon | analis | Spotfin Surfperch | Cool | Cool | Cool | Warm | Cool | Moderate |
| Paralabrax | maculatofasciatus | Spotted Sandbass | Warm | Warm | Warm |  | Warm | High |
| Sebastes | hopkinsi | Squarespot Rockfish | Eurythermal | Warm | Eurythermal | Warm | Warm | Low |
| Raja | stellulata | Starry Skate | Cool | Cool | Cool | Cool | Cool | High |
| Embiotoca | lateralis | Striped Surfperch | Cool | Cool | Cool | Eurythermal | Cool | Moderate |
| Rathbunella | alleni | Stripefin Ronquil | Cool | N/A |  | Cool | Cool | High |
| Sebastes | saxicola | Stripetail Rockfish | Eurythermal | Cool | Cool | Cool | Cool | Moderate |
| Embiotocidae | spp. | Surfperches | Warm | N/A |  | Cool | Eurythermal | Low |
| Cephaloscyllium | ventriosum | Swell Shark | Cool | Warm |  | Warm | Warm | Low |
| Platyrhinoides | triseriata | Thornback Ray | Cool | Warm |  | Cool | Cool | Low |
| Alopias | vulpinus | Thresher Shark | Eurythermal | Cool | Eurythermal |  | Eurythermal | Low |
| Galeorhinus | galeus | Tope Shark | Eurythermal | Cool | Cool | Eurythermal | Cool | Low |
| Atherinops | affinis | Topsmelt | Eurythermal | Cool | Eurythermal | Cool | Eurythermal | Low |
| Sebastes | serriceps | Treefish | Eurythermal | Warm | Warm | Warm | Warm | Moderate |
| Aulorhynchus | flavidus | Tubesnout | Cool | Cool | Cool | Cool | Cool | High |
| Sebastes | miniatus | Vermilion Rockfish | Cool | Cool | Cool | Cool | Cool | High |
| Hyperprosopon | argenteum | Walleye Surfperch | Eurythermal | Cool | Eurythermal | Warm | Eurythermal | Low |
| Atractoscion | nobilis | White Seabass | Warm | Cool | Warm | Warm | Warm | Moderate |
| Phanerodon | furcatus | White Surfperch | Eurythermal | Cool | Cool | Eurythermal | Cool | Low |
| Sebastes | entomelas | Widow Rockfish | Cool | Cool | Cool | Warm | Cool | Moderate |
| Anarrhichthys | ocellatus | Wolf Eel | Cool | Cool | Cool | Cool | Cool | High |
| Neoclinus | stephensae | Yellowfin Fringehead | Eurythermal | Warm | Warm |  | Warm | Moderate |
| Seriola | lalandi | Yellowtail | Warm | Cool | Warm | Warm | Warm | Moderate |
| Hermosilla | azura | Zebra Chub | Warm | Warm | Warm |  | Warm | High |
| Lythrypnus | zebra | Zebra Goby | Warm | Warm | Warm |  | Warm | High |
| Atherinops | affinis | Topsmelt | Cool | Cool | Cool |  | Cool | High |
| Clinocottus | embryum | Calico Sculpin | Cool | Cool | Cool |  | Cool | High |
| Rhinogobiops | nicholsii | Blackeye Goby | Cool | Cool | Cool |  | Cool | High |
| Gibbonsia | metzi | Striped Kelpfish | Cool | Cool | Cool |  | Cool | High |
| Gibbonsia | montereyensis | Crevice Kelpfish | Cool | Cool | Cool |  | Cool | High |
| Gobiesox | rhessodon | California Clingfish | Cool | Warm | Cool |  | Cool | Low |
| Hyposoblennious | gentilis | Bay Blenny | Eurythermal | Warm | Warm |  | Warm | Low |
| Hyposoblennious | jenkinsi | Mussel Blenny | Eurythermal | Warm | Warm |  | Warm | Low |
| Icichthys | lockingtoni | Medusafish | Eurythermal | Cool | Eurythermal |  | Eurythermal | Low |
| Liparis | callyodon | Spotted Snailfish | Cool | Cool | Cool |  | Cool | High |
| Liparis | mucosus | Slimy Snailfish | Cool | Cool | Cool |  | Cool | High |
| Rimicole | musacrum | Kelp Clingfish | Cool | Cool | Cool |  | Cool | High |
| Sebastes | flavidus | Yellowtail Rockfish | Cool | Cool | Cool |  | Cool | High |
| Sebastes | serranoides | Olive Rockfish | Cool | Cool | Warm |  | Cool | Low |
| Stellerina | xyosterna | Pricklebreast Poacher | Cool | Cool | Cool |  | Cool | High |
| Ulvicola | sanctaerosae | Kelp Gunnel | Cool | Warm | Warm |  | Cool | Moderate |
| Xiphister | atropurpureus | Black Prickleback | Cool | Cool | Cool |  | Cool | High |
| Apodichthys | fucorum | Rockweed Gunnel | Cool | Cool | Cool |  | Cool | High |
|  |  |  |  |  |  |  |  |  |
