# Supplementary material for: Species-specific thermal classification schemes can improve climate related marine resource decisions
Source: PLoS One. 2021 Apr 28;16(4):e0250792. doi: 10.1371/journal.pone.0250792 (PMC8081253; doi:10.1371/journal.pone.0250792)
Supplement: Attachment — Submitted filename: PONE-D-20-36102_reviewer.docx [file pone.0250792.s003.docx]

Dear authors and editor,

I enjoyed reading “Species-specific thermal classification schemes can improve climate related marine resource decisions”. I personally find predictions about the effects of warming based on species being cold or warm adapted vague, partly because classifications do not seem robust and often seem based on simplistic views of a species. This is a neat paper that address just that uncertainty, by developing a classification scheme for the thermal affinity of fish using multiple data sources (including expert opinion) in order to robustly classify species as either “warm“ or “cold”. Next, differences in the response of these two thermal groups to a marine heatwave, acting as a natural experiment, are evaluated. I have a few major and some minor comments that I hope the authors find useful.

**Major comments**

- Line 84: I was wondering if you had discussed the observation that that warm-adapted/tropical species have narrower thermal tolerances that sup-tropical or temperature species. This would suggest that cold/temperate species might actually be better to withstand heatwaves because of their wider thermal windows than warm/sub-tropical/tropical species who live closer to the “edge”. In other words, what matters for a species ability to survive a heatwave is more linked to the thermal width rather than at which temperature the preferred temperature is. Is this something that also applies to your species, or would you say that all your species have more or less the same thermal width?
- A follow up question related to the natural experiment that is the heatwave: is the density a good metric in this case? I think this depends on what drives the fluctuations in abundance of these species in this system. You only use two years (2012-2013 and 2014-2015). The densities in the heatwave years are likely very dependent on the recruitment in the normal years. Basically, what is driving the increase in density of the warm species in the warm period? Larger recruitments? If so, are fish recruited in the two-year period sampled by the survey in the same period, or are they too small? Or is that warm species are moving into the area because it’s warmer? Do you expect a delayed response in density from a heatwave? (Same goes for the difference in cold species density but opposite). Essentially, could you explain a little bit what the relevant mechanisms are?
- Did the heatwave act uniformly across the whole study area? Because you use density below and above Point Conception to characterize species in one of the methods, I guess it’s important to state explicitly that that the heatwave acted equally above and below Point Conception, else it could be a confounding effect.
- Line 182: The approach to evaluate the responses seems too simplistic and descriptive. I see that the average changes are very clear (by thermal classification), but nothing about the uncertainty. Have you considered fitting a regression to this? For instance, for each species, evaluate if there’s a difference in density between cold and warm years, and then see if there’s an effect of the thermal classification (or set it up in some other way). Because with all species pooled, I can’t tell if the large changes are driven by a few species or a little bit by all species in groups. Or even if or how many of the warm species show declines in densities in the heatwave, and vice versa. Also, it would be interesting to see if the response was clearer for species with better agreement across classification methods (higher rank). Without these analyses, it’s very difficult to assess how statistically clear the findings are.
- Line 141: I wonder if it’s interesting to actually look at the eurythermal species, kind of as a control. You would expect them to be somewhere in between the cold and warm species in terms of changes in density, right?

**Minor comments**

- Line 44: “natural resources”
- Line 127: A map of the area would have been nice for readers who are not familiar.
- Line 157: Where the experts instructed to not google species or use FishBase? Otherwise, data such as thermal ranges would be easy to look up. Where the classification from source 3 and 4 more similar, for instance?
- Figure 1: see major point about the analysis. I think it would be great to analyze this differently to get a feeling for the statistical uncertainty and the distributions of species responses.
- Figure 2: needs to be explained more. I think it’s best to spell out what CTI stands for here since it’s only mentioned once in the text (and there it can be explained, not just mentioned). I don’t really understand what the y-axis represents in the right graphs. What is meant by management here? Is it something that benefits or aims to protect the population here, or relaxed management as in fewer rules leading to higher exploitation? And how is it related to the left graph?
